# Paediatric Multiple Sclerosis: A Scoping Review of Patients’ and Parents’ Perspectives

**DOI:** 10.3390/children9010011

**Published:** 2021-12-25

**Authors:** Maria Luca, Nerea Ortega-Castro, Francesco Patti

**Affiliations:** 1 Centre for Addiction, Via Pò 2, 95031 Adrano, Italy; 2 Psychology Department, Loyola University, 41704 Seville, Spain; nerea.ortega@deusto.es; 3 Department “GF Ingrassia”, Section of Neurosciences, University of Catania, 95125 Catania, Italy; patti@unict.it

**Keywords:** paediatric multiple sclerosis, patients, parents, perspectives, review

## Abstract

Dealing with paediatric-onset multiple sclerosis is particularly challenging for the young patients and their families, due to its unpredictable symptoms and uncertain outcome. This review aimed at synthesising the qualitative evidence regarding the perspectives about paediatric-onset multiple sclerosis, as expressed by the patients and/or their parents. A literature search was conducted on PubMed and CINAHL. The advanced multi-field search allowed to perform an abstract/title search in both databases, using keywords, combined through Boolean operators. Additional search strategies were adopted: searching the reference list of the selected papers; searching for key authors in the field. All the relevant papers were thoroughly revised using The Joanna Briggs Institute’s data extraction form for qualitative evidence as a guidance. Eight papers were selected. The analysis of these papers allowed to identify some common issues pertaining paediatric-onset multiple sclerosis: (1) onset of symptoms, (2) diagnostic process, (3) reaction to the diagnosis, (4) management and acceptance of multiple sclerosis. The burden of multiple sclerosis was confirmed. However, the young patients and their parents can adjust to the disease. Both the community and the health care professionals must strive to prevent the families dealing with multiple sclerosis from experiencing solitude and rejection.

## 1. Introduction

### 1.1. Paediatric-Onset MS

Multiple sclerosis (MS) is a demyelinating disorder that causes the destruction of the myelin sheath around the nerve fibres. The aetiology of the condition is not fully known, but autoimmune mechanisms are universally recognized as relevant [1,2].

MS can affect a variety of functions, thus causing heterogeneous symptoms, many of which are highly a-specific [3]: weakness, impaired ambulation, reduced vision, difficulties in maintaining balance, sexual dysfunctions, etc. Apart from the physical symptoms, cognitive-behavioural disturbances (impaired memory, reduced speed of information processing, depression, bipolar disorder, to name just a few) are not uncommon [3].

Fatigue, namely an excessive exhaustion when performing usual activities, is an extremely common (and rather uncontrollable) symptom, that should be distinguished by fatigue reported in other chronic conditions, being able to affect sleep, mood and general well-being [4]. There are different phenotypes of MS, but the relapsing-remitting (RR) one is the most frequent, accounting for nearly the totality of MS cases. As suggested by the name, RR-MS is characterized by an alternation of relapses (new or worsening symptoms, lasting more than 48 h, impairing one or more functions) and periods of remission, namely relative stability [5]. Unfortunately, there is no way to accurately predict the number of relapses and the general course of the disease, the latter being characterized by high levels of uncertainty and heterogeneous features among the affected people. As a matter of fact, uncertainty itself is a major cause of distress in the MS population, causing anxiety, depression and worrying thoughts. Indeed, people suffering from MS deal with a peculiar disease, causing “strange” symptoms and burdened by an unpredictable outcome [6,7]. However, it should be noted that the disease-modifying therapies (DMTs), such as fingolimod, natalizumab and rituximab, may lead to a better disease control and a reduced relapse rate in paediatric MS [8].

Nonetheless, it cannot be denied that chronic medication is troublesome. More specifically, many pharmacological agents (i.e., interferon-beta, typically used as first-line treatment) are administered through injections and can frequently cause minor side effects (swelling at the injection site, fever). Less frequently, the side effects may be potentially life-threatening, due to organ toxicity and severe infections [9]. Hence, if on the one hand MS may limit everyday activities due to functional impairment, medication certainly represents an additional problem, due to the commitment to a certain administration schedule, the management of the side-effects and the stigmatizing reality of being under a chronic treatment [10].

The disabling nature of MS, in fact, cannot be reduced to the physical symptoms, but is largely related to the psycho-social consequences of the disorder, along with the financial ones (absenteeism, loss of job, inability to work) and, last but not least, the consequences of MS on the significant others [11,12]. Indeed, the uncertainty for the future, particularly when MS occurs early in life, threatens many of the dreams and projects we normally have, such as living alone, finding a job, getting married, having children [13]. In addition, persons with MS frequently experience feelings of hopelessness [14], the latter representing not only a psychological reaction to MS, but a possible symptom, due to its organic correlates (e.g., reduced metabolic activity in the ventromedial prefrontal cortex).

These aspects cannot be underestimated, since MS is a rather frequent disorder, affecting 2.8 million people worldwide [15]. The condition typically affects young adults (20–40 years old), so that paediatric onset (less than 16 or 18 years old) is relatively rare, accounting for the 5% of all MS cases [16]. However, it should be noted that paediatric MS is now recognised with increasing incidence [17]. 

### 1.2. Diagnosis of MS

From a diagnostic point of view, regardless to the age at onset, the most important tool for the diagnosis of MS is magnetic resonance imaging (MRI). For children ≥12 years old, after the onset (attack), the evidence of new T2 lesions or gadolinium-enhancing lesions (indicating active disease), separated by a period of at least 1 month, confirm the diagnosis. For patients younger than 12, at least two clinical events must occur before the diagnosis can be formulated [16]. From a radiological point of view, children’s and adolescent’s MS has some peculiarities, such as the higher lesion load and the marked inflammatory pattern, despite lesions being usually less destructive and more likely to recover with time [18].

Despite the advancements in the field, a delayed diagnosis of MS, not uncommon among adults, is sadly highly frequent among children, due to the need to exclude other, more frequent, neurologic conditions (e.g., clinically isolated syndrome, acute disseminated encephalomyelitis). Moreover, not all paediatricians are able to perform a correct diagnosis and may unnecessarily postpone a referral to specialist centres. For the sake of completeness, it should be noted that even neurologists may not have expertise regarding paediatric MS, due to its relative rarity [19]. In addition, the very onset of the disease (strange symptoms that last few days and then disappear) is highly confusing for the parents [20] who may misinterpret their children’s complaints as “whims of adolescence” (e.g., fatigue interpreted as laziness). The delay of the diagnosis adds stress to the already scared patients and families [21], along with increasing the risk for a more severe outcome, due to a later treatment initiation, also considering that the prognosis of paediatric-onset MS is generally worse than adult-onset MS [16]. In addition, the disease course of paediatric-onset MS is characterized by a higher relapse rate [22]. In general terms, more MS relapses may lead to limited recovery of function, as reported in a study relating higher relapse rates to poorer recovery after optic neuritis, namely the inflammation of the optic nerve that affects visual acuity [23].

When, finally, a diagnosis is reached, its delivery to the family comes as a shock [20]. Indeed, if adjusting to MS is difficult for adults and their families [12], let us imagine how devastating it may be to receive such a diagnosis at an early age, not only for the young persons with MS, but also for their parents, who may feel the burden of helping their children at all costs, advocating for them and taking decisions regarding treatment, while facing a rather uncontrollable disease.

### 1.3. Burden of Paediatric MS

There is an extensive body of quantitative evidence highlighting that MS may threaten both the patients’ and their caregivers’ well-being, also depending on the level of accumulated disability [24,25,26]. However, it cannot be neglected that the caring experience lived by the families can be a powerful force towards adjustment and should be more valued by the physicians [27,28]. As a matter of fact, taking care of a person with MS does not entail only negative, but also positive aspects [29]: discovering personal and family resources, embrace change and apply coping strategies, such as supportive engagement and positive reframing. Based on that, enhancing the well-being of the whole family facing MS could result in a more successful adjustment to the disease [28].

These aspects acquire a crucial importance when paediatric-onset MS is considered. In fact, it is easy to envision how a chronic and unpredictable illness occurring in a delicate life period (childhood or adolescence) may require a double effort to the young patients (e.g., dealing with the emotional problems typical of adolescence, *plus* MS) and their parents (stressors related to parenting, *plus* caring for a person with MS).

This paper builds upon two theoretical frameworks: the peer–grief dynamics model [30] and the resiliency model of family stress, adjustment and adaptation [31].

The peer–grief dynamics model, theorized by Thannhauser [30] to explain the psychosocial experiences of young persons with MS, highlights that these patients experience a process of grief, whereby recurrent losses (of health, control, identity, normalcy, etc.) are accompanied by a variety of manifestations, such as anger, depression, denial and self-isolation. The response of their peers to the disease may constitute a further loss or facilitate the process of acceptance. Despite providing a theoretical framework regarding the specific population of young MS patients, the grief-peer dynamics model “overlooked the role of the family in the experiences of adolescents with MS” [30], p. 775, as stated by Thannhauser herself.

As a matter of fact, there already is a framework that could account for the families’ response to major stressors, such as that of receiving a diagnosis of juvenile chronic illness, namely the resiliency model of family stress, adjustment and adaptation. According to this model, when a family faces a stressor, it appraises the problem and, then, puts into place a variety of resources, in the attempt to maintain its functioning. The support from the community, the specific abilities of the family members, the problem-solving and coping strategies are examples of resources that may “save” the family’s normalcy [31].

In light of what has been discussed so far, shedding light on the perspectives about paediatric-onset MS as directly expressed by the patients and their parents may be useful to guide the healthcare professionals’ work and relational style when approaching the families.

Indeed, it is easy to hypothesise that if the family as a whole does not successfully elaborate and acknowledges the losses caused by MS, the adaptation to the disease might be at risk, along with the quality of life of all the people involved. In other words, when dealing with major stressors, such as paediatric-onset MS, the whole family, and not only the patient, must be the focus of the health care professionals’ attention. More knowledge on the subject means more support for the families, and may result in a better family-physician relationship, ultimately leading to a better disease management. Building on this, this scoping review aimed at synthesising the qualitative evidence regarding the perspectives about paediatric-onset multiple sclerosis, as directly expressed by the patients and/or their parents.

## 2. Materials and Methods

A literature search was conducted on PubMed and CINAHL, separately used. The search strategy was consistent among the databases. More specifically, the advanced multi-field search allowed to perform an abstract/title search in both databases, using the following search terms, combined through Boolean operators (AND, OR) as needed:
“paediatric” OR “pediatric” OR “child” OR “children” OR “childhood” OR “juvenile” OR “youth” OR “youngster*”“multiple sclerosis” OR “MS” OR “demyelinating disorder” OR “relapsing-remitting”“perspective*” OR “perception*” OR “opinion*” OR “experience*” OR “attitude*” OR “impression*” OR “lived experience”“caregiver*” OR “parent*” OR “mother*” OR “father*” OR “family”

The asterisk (*) operator was used to find words with the same stem and different endings (i.e. youngster, youngsters). According to the settings specific of each database, the keywords were combined (AND) into a single literature search. In order to maximise the retrieving of papers, additional search strategies were adopted: searching the reference list of the papers included in the review; searching for key authors in the field.

### 2.1. Selection of the Papers

Inclusion criteria: original qualitative studies (of any type); written in English; any date of publication; specifically investigating the perceptions, perspectives, lived experience regarding any aspect related to paediatric multiple sclerosis, among the affected children/young adults with a clear paediatric onset and/or their parents; any sex and ethnicity. Review papers, theoretical papers, editorials and grey literature (papers and materials available outside the traditional academic publishing, e.g., congress proceedings, dissertations) were excluded from the review.

### 2.2. Relevance

**Figure 1:**
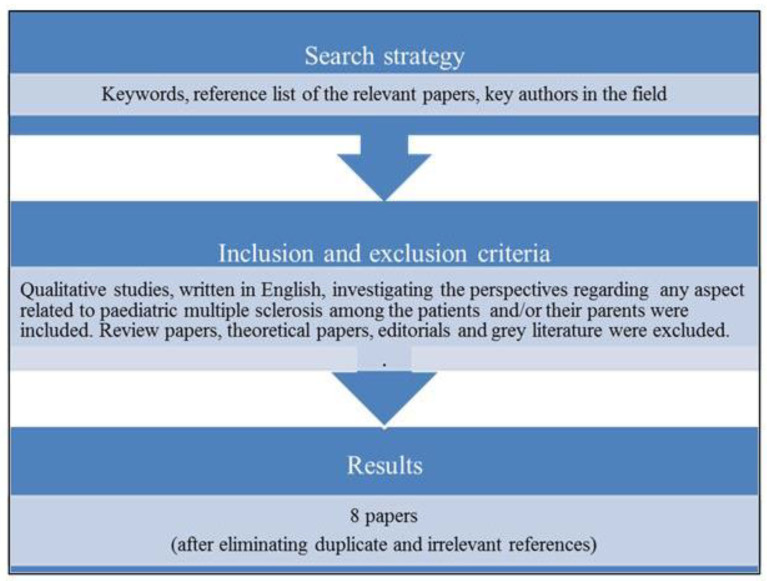
Review process.

All the papers meeting the inclusion criteria were considered as relevant to the review. A first screening of eligibility has taken place considering the titles and abstracts of all the papers retrieved through the literature search. Then, the full texts of the potentially eligible papers have been accessed, thus reaching a definite decision of inclusion/exclusion. Duplicate references were eliminated. See Figure 1 for a visual presentation of the review process.

### 2.3. Data Extraction

The current study is a scoping review applying a descriptive synthesis of qualitative evidence. Hence, all the relevant papers were thoroughly revised in order to extract the information contributing in answering the review question. The Joanna Briggs Institute’s [32] data extraction form for qualitative evidence was used as a guidance for the data extraction phase. The form considers several aspects, such as the topic considered, the setting, the population and the main conclusions [32].

## 3. Results

The search strategy (databases, references of the selected papers, search of key authors in the field), once the duplicate references had been eliminated, allowed to retrieve 11 possibly relevant papers. Eight of them, published between 2005 and 2019, met the inclusion criteria.

Five out of 8 papers applied a grounded theory approach, 2 used thematic analysis and 1 adopted a phenomenological perspective. The studies were performed in different countries: USA, UK and Canada. Three studies were conducted relying on a single centre, the others referred to more than one clinic, to NHS trusts, MS organisations and/or online support fora. Seven out of 8 papers used in-person interviews as a method of data collection. One paper involved patients interviewed by telephone [33]. Two papers [30,34] report the use of other strategies alongside interviews (blogs or focus group).

Overall, the focus of all papers was that of gaining insight on the lived experience of persons with paediatric onset MS and/or their parents. Some of them gave special attention to specific issues, such as fatigue [35], adjustment to MS [34], diagnostic process [19,33], peer relationships and their role in influencing the grieving process subsequent to receiving the diagnosis and dealing with MS [30].

Considering the relevant papers as a whole, 152 parents and 61 patients provided their unique experience on paediatric-onset MS. Despite all studies including patients with clear paediatric-onset MS, only one research actually collected data from patients <18 years old [19]. For a more detailed overview of the selected papers, please refer to Table 1.

All the selected studies (each one through its peculiar data analysis) report a number of categories, themes and/or subthemes representing the issues arising from the interviews and solicited by the participants’ own words. For a summary of the main themes identified in each paper, refer to Table 2, Table 3, Table 4 and Table 5 (please note that the Tables have the only purpose to summarize the content of the relevant papers. By no means the Tables represent the authors’ actual point of view or intend to appropriate their intellectual property. Hence, the reader is invited to refer to the original articles).

Regardless to the specific themes identified in each paper, the whole body of selected literature allows to identify some key aspects which seem to be rather common when the accounts of the young persons with MS and their parents are taken into consideration. The following paragraphs provide a detailed description of the main issues identified when considering the papers analysed in this scoping review, namely: (1) onset of symptoms, (2) diagnostic process, (3) reaction to the diagnosis, (4) management and acceptance of MS.

### 3.1. Onset of Symptoms

The symptoms of MS are variable, but they frequently involve the visual and sensitivity functions. For the very nature of MS, the symptoms typically come and go, so that the patients may feel that everything is back to normal. The fear related to the occurrence of strange and disabling symptoms, as well as their tendency to suddenly disappear, may favour a “wait and see approach” [19], whereby the avoidance of symptoms may even represent a defence mechanism against the scary reality [34].

Many symptoms may be overlooked and mistaken for whims of adolescence so that, when the diagnosis is finally reached, parents may feel guilty for having misinterpreted the early signs of MS [20].

### 3.2. Diagnostic Process

When the family finally seeks medical attention, the diagnostic process proves itself long and frustrating. Some patients and parents are also put in the position of being questioned and even blamed of “faking” (or overreacting to) the symptoms, the latter being frequently re-conducted to psychosocial problems or viral infections [19,34]. The diagnostic uncertainty is further confirmed by the fact that most patients received 2 or more alternative diagnoses before that of paediatric-onset MS. In this confusing context, doctors are frequently perceived as rather unprepared and/or unwilling to formulate the diagnosis, some of them even stating that MS is a disease unique to adults [19,21,33,34].

Once the diagnosis has been confirmed, parents usually appreciate when doctors use simple terms, maintain a hopeful position (while avoiding unnecessary sugar-coating), inform them without the child being present and allow them to decide how the news should be delivered to their child [20,33].

### 3.3. Reaction to the Diagnosis

The selected papers consistently report that receiving the diagnosis determines a reaction of fear, sadness and shock. Notably, due to the long and scary diagnostic process, it may paradoxically come as a relief: once the unknown has been labelled, appropriate action may be taken [20,33,36]. The young patients sometimes display an apparent carelessness, interpreted by their parents as either denial or expression of their resilience [20].

Nonetheless, the patients usually feel overwhelmed as well, also contemplating the possibility of dying because of MS, interpreting it as a potentially fatal condition [36]. Their mood swings and the vortex of emotions (anger, sadness, irritability, anxiety, depression) experienced by them reminds the “classic” grief experience [30].

### 3.4. Management and Acceptance of MS

All the papers addressing the management of MS in the interviews solicit the feeling that both the young patients and their parents are engaged in an iterative process between suffering and acceptance. Indeed, MS is burdened by psychological, social, physical and even financial consequences [20], the latter related to the costs for travelling or even medication, depending on the health care policy of the country. The young persons with MS endure a series of failures, due to the considerable impact MS has on school performance, quality of the peer relationships and parental well-being [36]. As a matter of fact, paediatric-onset MS goes even further than stealing normalcy from the families. Indeed, occurring in a really delicate and crucial period of life, it determines a shift in the identity of the affected persons, who abruptly see themselves as “ill”. Another serious consequence of MS at such a young age is the loss of hope, plans and dreams for the future, due to the unpredictable nature of the condition [30].

Uncertainty becomes an unwelcome companion of the families dealing with paediatric-onset MS, characterizing the course of the disease from the very early manifestation, to the difficult-to-reach diagnosis and, last but not least, the unpredictability of symptoms, relapses and prognosis [21].

Adjusting to the medication schedule, facing the cognitive impact of MS, re-consider future plans, deal with the side effects of the medication, in other words plan all your life around MS, represent challenges unique to the families dealing with this condition [20].

As if this was not enough, a considerable number of persons with MS, including children and adolescents, have to deal with fatigue, that cannot be interpreted as “simple tiredness”, since it is a condition leading to both mental and physical exhaustion [4,35]. The young patients are well aware of the stigmatising effects of fatigue. Indeed, fatigue their participation to peer activities. Moreover it provokes feelings of guilt, while causing worries about being treated differently by other people [35]. Moreover, since its causes are largely unknown, fatigue puts the parents in the distressing position of having to distinguish fatigue from adolescent’s behaviour [35].

Despite dealing with MS entailing a great effort, many families adopt a variety of strategies aimed at “moving on”. Recurrent accounts refer to the need of maintaining a positive thinking [21,36] and focus on the present [20]. It is worth considering that MS can even represent a transformative experience, teaching the young patients what really matters in life, pushing them to fight against the adversities and even contribute to their development into more compassionate and mature human beings, able to advocate for self [30,34].

A positive force towards adjustment is represented by social support. Thannhauser’s studies [30,34] highlight the role of peer relationships in favouring either the suffering experience or disease acceptance, according to the negative versus positive reactions to the disease and to what it entails (e.g., medication). More specifically, the young patients may lose their friends due to the disease, thus being exposed to social isolation. On the other hand, the deepening of old friendships and the creation of new, supportive ones, represent powerful incentives towards adjustment [30,34]. Other papers also refer to social support as a fundamental resource for the families dealing with paediatric-onset MS [20,36].

Unfortunately, disclosing the diagnosis to others does not always lead to a positive outcome. Indeed, the families are exposed to negative thinking and doubts about the diagnosis [21], while the young patients are exposed to stigma, teasing and rejection [34]. As a result, the need to be cautious and selective when choosing whom to tell is a common theme in the families’ accounts [20,21,30,35,36]. The disclosure of the diagnosis may even represent a way to test the value of new relationships [36]. In addition, despite entailing some risks, disclosing the diagnosis could represent another step towards acceptance [30].

Although some families do not engage in the MS community in order to protect their children from seeing severe cases, such organisations have been reported to provide substantial support, even financial one, when needed. Hence, some parents recommend these resources and encourage physicians to provide a list of resources when the diagnosis is reached [20,33].

Overall, not only the support from significant others, but also the actual management of the disease could contribute to the process of acceptance. Indeed, seeking information, adopting a healthier lifestyle [21], managing the medication, developing emotional awareness and interpreting MS as something that does not define the self [35] represent empowering experiences eliciting a sense of control when facing the uncertain.

## 4. Discussion

The methodologies adopted by the selected papers (grounded theory, thematic analysis and phenomenology), consistently with the principles valued by qualitative research, produced an in-depth understanding of the families’ lived experience of MS, its symptoms, and its management. Overall, the selected papers reach a relatively big sample size (152 parents and 61 patients), also considering that paediatric-onset MS is not a frequent condition. However, it must be noted that qualitative research does not value sample size in terms of quantity, but in terms of thematic saturation, implying that the number of participants must be dictated by their contribution to the generation of new themes and insights pertaining the phenomenon of interest [37].

The selected articles, each one with its own specific focus, allowed to obtain an overview of the unique challenges experienced by the families dealing with paediatric-onset MS, as well as the resources these family value as facilitators of adjustment. For their very nature, qualitative studies do not usually enlist generalizability (to settings other than those characterizing the original research) among their main strengths. However, some findings may be broadly transferable to other similar contexts [37].

Considering the aspects discussed in this thesis, adjusting to medication, losing friends, dealing with prejudice, facing functional limitations and uncertain future are experiences common to patients and parents dealing with other paediatric-onset chronic conditions, such as idiopathic arthritis [38,39], chronic renal failure [40] and psoriasis [41].

The articles deemed relevant to this scoping review have their own limitations, particularly those (the majority) involving adult patients (even if with a clear paediatric-onset), thus risking recall bias. However, it is fairly conceivable that experiences with marked consequences on one’s own life and deeply affecting one’s own emotional status (such as that of receiving a diagnosis of paediatric-onset MS) are not easy to forget, as theorized by neuroscientific papers [42].

The whole body of literature (papers deemed relevant to this scoping review) allowed to identify some core issues related to paediatric-onset MS, which have been summarised as: onset of symptoms, diagnostic process, reaction to the diagnosis, management and acceptance of MS. Overall, all the analysed papers, including the only one involving patients <18 years old [19], highlight uncertainty as a core issue when dealing with paediatric MS, with diagnosis being a considerably painful process. The results of this review are in line with the hypothesis that families facing the diagnosis of MS undergo a complex process of change and adaptation. This review contributes to current knowledge, shedding light of the lived experience of families dealing with paediatric-onset MS, ultimately allowing to answer the review question: “what are the perspectives about paediatric onset multiple sclerosis among the patients and their parents”?

The findings reported in this paper may be of use in clinical practice. In fact, since recognising the needs and challenges of the families, as well as the barriers and facilitators of adjustment, could inform and improve how the health care professions approach these families, in order to avoid further (and highly unnecessary) burden to them. Indeed, a tailored support may favour the young persons with MS and their families to transition towards acceptance, the latter resulting in a more effective (and less-stressful) management of the disease. A better physician-family relationship may also favour the early initiation of treatment, in order to reduce the relapse rate. Interestingly, the control of relapses may acquire further importance in the COVID-19 pandemic era, in light of the speculation (to date disconfirmed) of a higher risk of exacerbation following contraction [43].

## 5. Conclusions

This scoping review allowed the identification of core issues pertaining paediatric-onset MS. The physical, psychological, social and financial burden of the disease was confirmed. However, both the young persons with MS and their parents have the potential to adjust to the disease. Being social support a powerful force towards acceptance, both the community and the health care professionals must strive to prevent the families dealing with MS from experiencing solitude and rejection. Despite the limitations stated above (recall bias), the findings of this review highlight the struggles and needs of families dealing with paediatric-onset MS. Further studies on the subject are needed, in order to expand on the aspects discussed in this paper.

## Figures and Tables

**Table 1:** Overview of the selected papers (content guided by The Joanna Briggs Institute, 2020).

Papers (Titles Abbreviated)	Methodology	Methods	Phenomenon of Interest	Recruitment	Participants	Country	Data Analysis
Families’ experience of pediatric onset multiple sclerosis. Cross et al., 2019	Thematic analysis	In person interview	(1) Stresses of parenting a child with MS (2) Stress management	2 MS centres for children	19 parents 2 couples	USA	Identification of themes
Insights and recommendations from parents receiving a diagnosis. Hebert et al., 2019	Thematic analysis	Phone interview	Receiving the diagnosis	National MS Society	42 parents	USA	Identification of themes
Living with uncertainty and hope: a qualitative study exploring parents’ experiences. Hinton et al., 2017	Grounded theory	In person interview	Experiences of parents dealing with paediatric MS	16 MS centres 4 voluntary organisations	31 parents	UK	Coding and generation of categories using NVivo software
“It feels like wearing a giant sandbag”. Adolescent and parent perceptions of fatigue. Carroll et al., 2016	Elements of grounded theory	In person /Phone interview	(1) Experiences of fatigue in patients and parents (2) Management of fatigue	NHS paediatric neurology clinics MS charities Support fora	15 patients 13 parents Age: 6–18, reporting fatigue	UK	Coding Development of themes and sub-themes
Paediatric multiple sclerosis: a qualitative study of families’ diagnosis experiences. Hinton et al., 2015	Elements of grounded theory	In person interview	(1) Diagnostic process (2) Families’ support needs (3) Barriers/facilitators to early diagnosis	16 NHS Trusts 4 voluntary organisations	31 parents 21 patients Age: 8–17	UK	Identification and comparison of codes using NVivo software
Navigating life and loss in pediatric multiple sclerosis. Thannhauser, 2014	Charmaz’s constructivist grounded theory	(1) In person interview (2) Blog	Adjustment to paediatric MS	3 MS centres	7 patients 6 parents Age: 16–21	Canada	Coding and generation of categories consider that reality is socially constructed
Grief-peer dynamics: understanding experiences with pediatric multiple sclerosis. Thannhauser, 2009	Grounded Theory	(1) In person interview (2) Focus group	(1) Experience of adolescents with MS (2) Peer relationships within this experience	1 MS centre	6 patients 6 parents Age: 14–21	Canada	Coding Generating of categories Finding relationships within the data Theorizing
Experiences of children and adolescents living with multiple sclerosis. Boyd et al., 2005	Phenomenology	In person interview	Experiences of children and adolescents living with MS	1 MS centre	12 patients Age: 8–18	Canada	Narrative analysis. Themes were compared and integrated using NVivo software

**Table 2:** Summary of the themes identified in Cross’s and Hebert’s studies.

Papers (Titles Abbreviated)	Focus	Identified Areas of Interest
Families’ experience of pediatric onset multiple sclerosis. Cross et al., 2019	(1) Stresses of parenting a child with MS (2) Stress management	*Experience prior to the diagnosis*: shock and fear for the sudden symptoms; shame and guilt for dismissing the early signs *Receiving the diagnosis*: praise for physicians clarifying that prognosis is not necessarily dreadful and there are new therapies for paediatric MS *Reaction to the diagnosis* (parents): shock and fear Children’s reaction (parents’ account): from severe distress to apparent carelessness (The reaction to the diagnosis entailed decisions about disclosing MS to others) *Emotional impact of paediatric-onset MS:* anxiety (relapses, progression…) responsibility (genetic contribution) need to hide negative emotions from children Emotional impact in children (parents’ account): fear, frustration, social isolation, no distress (denial or resilience?) *Treatment:* concerns about efficacy and side effects privacy (bruises at the site of injection) needle phobia Children usually involved in decision-making *Impact at school:* cognitive impairment absenteeism (hospitalization, symptoms…) variable school support friendships usually preserved Family life: perform the injection for their children assistance during hospitalization deal with information, costs and paperwork putting job at risk neglecting the spouse and the other siblings *The Multiple Sclerosis Community:* variable level of engagement and perception of usefulness praise for informational and financial support Living with paediatric-onset MS: maintaining the quality of life setting reasonable expectations for the future balancing independence and vigilance *Concerns for the future:* children losing independence parents not being able to take care of their child conducting a normal life effectiveness and affordability of the medications maintaining a healthy lifestyle *Advice for other parents:* find social support focus on the present talk with doctors and involve your child engage with the MS community
Insights and recommendations from parents receiving a diagnosis. Hebert et al., 2019	Receiving the diagnosis	*Initial symptoms and diagnostic process:* common presenting symptoms: visual disturbance; numbness and or/tingling of the extremities many children receiving 2 or more diagnosis prior to MS symptoms frequently dismissed as signs of infection, anxiety, ailment *Parents’ initial reaction to the diagnosis:* scared and overwhelmed; shock; relief (for knowing) *Recommendations from parents to medical professionals:* increase knowledge of paediatric MS within the medical community let parents decide how the diagnosis is delivered to their child listen to parents, because they know their child provide resources to families (informative material, support groups, etc.)

**Table 3:** Summary of the themes identified in Hinton’s and Carroll’s studies.

Papers (Titles Abbreviated)	Focus	Identified Areas of Interest
Living with uncertainty and hope: a qualitative study exploring parents’ experiences. Hinton et al., 2017	Experiences of parents dealing with paediatric MS	*Diagnostic uncertainty:* delayed diagnosis and doubtful professionals increased fear *Daily uncertainty:* symptoms are unpredictable difficulty in anticipating the child’s needs lack of information on how to manage the disease on a daily basis *Interaction uncertainty:* people (even doctors) doubt the diagnosis once disclosed by the parents people do not believe that apparently healthy children have special needs *Future uncertainty:* unknown future for their child *Strategies to manage uncertainty:* information searching (potentially distressing when facing professionals’ limited knowledge and other patients’ negative outcome) continuous monitoring (try to identify early signs of a relapse; difficulties in distinguishing adolescents’ behaviour and MS symptoms) implementing changes (healthy lifestyle, without being certain of its efficacy) optimistic thinking (prognosis may be good, avoid people with negative thoughts or severe cases, live the present)
“It feels like wearing a giant sandbag”. Adolescent and parent perceptions of fatigue. Carroll et al., 2016	(1) Experiences of fatigue in patients and parents (2) Management of fatigue	(1) Lived experience and impact of fatigue fatigue is physically and mentally exhausting fatigue affects school fatigue affects family plans (2) Uncontrollability and uncertainty fatigue is uncontrollable (some fight it, others accept it) the causes of fatigue are uncertain (parents may not distinguish between teenage and MS fatigue) (3) Finding a balance between pushing yourself and resting too much (most parents accept excessive resting) (4) Concern (parents): fatigue negatively affecting mental health, making the patients feel inferior, becoming more disabling when facing adult life, stealing important experiences(5) Social support and disclosure (patients): fear of being treated differently, feelings of guilt towards friends. Those who disclosed usually received support

**Table 4:** Summary of the themes identified in Hinton’s and Thannhauser’s studies.

Papers	Focus	Identified Areas of Interest
Paediatric multiple sclerosis: a qualitative study of families’ diagnosis experiences. Hinton et al., 2015	(1) Diagnostic process (2) Families’ support needs (3) Barriers/facilitators to early diagnosis	*Symptoms:* gradual onset of a-specific symptoms, that come and go *Recognising a problem:* “wait and see approach” *Seeking medical advice:* general practitioner first *Communicating concerns to medical professionals:* from being heard to being blamed of imagining symptoms, overreacting to/causing them *Medical interpretation of the symptoms: * In primary care, symptoms frequently dismissed as signs of viruses or psychological problems. In secondary care, paediatricians seemed to be unprepared to interpret MRI findings. *Questioning medical opinion:* frustration when symptoms are dismissed patients’ accounts of uncertainty parents taking the lead during the visits parents’ concerns over their credibility *Receiving a diagnosis of MS:* neurologists sometimes reluctant to diagnose MS praise to clinicians using simple terms uncertainty towards the diagnosis, due to conflicting medical opinion uncertainty may increase hope (patients may improve in the future)
Navigating life and loss in pediatric multiple sclerosis. Thannhauser, 2014	Adjustment to paediatric MS	*Recurring loss in the patients * (1) suffering: shock, confusion, sadness (2) fearing the unknown (loss of future) (3) losing trust (professionals’ uncertainty, being blamed of faking the symptoms) (4) sense making (reconstruction of the believes about themselves and reality) Denial as a defence mechanism. *Carrying on in the patients * (1) becoming me: knowing more about yourself (2) putting MS in its place: MS dose not define the self (3) pushing boundaries: risk-taking behaviours aimed at reducing the limitations imposed by MS (4) finding normal: maintaining normalcy and reinventing normal (re-conceptualization of independence) (5) becoming an expert: controlling symptoms through lifestyle; making medical decisions by themselves; information seeking (severe cases increase suffering); advocating for self (setting boundaries with others); planning for the future (e.g., intellectual career) *Selectively disclosing:* needing to know (inform people at school when symptoms are affecting these areas) wanting to tell (disclose to significant others to receive support) *Meaning making: * integrating MS in one’s own life through perspective taking, reprioritizing, finding purpose, remaining hopeful TURNING POINTS The following aspects strongly influence the experiences of loss and the ability to carry on: (1) labelling the disease; (2) develop emotional awareness; (3) managing medication; (4) dynamic relationships (support from others help to carry on; losing experiences and friends, being teased and rejected contribute to grief)

**Table 5:** Summary of the themes identified in Thannhauser’s and Boyd’s studies.

Papers	Focus	Identified Areas of Interest
Grief-peer dynamics: understanding experiences with pediatric multiple sclerosis. Thannhauser, 2009	(1)Experience of adolescents with MS (2)Peer relationships within this experience	*Grief experience * Mood swings; shifts between grief and acceptance Major loss: physical health Secondary losses: identity shift; loss of control due to unpredictable symptoms; loss of friends; loss of hope for the future; loss of normality (medication, diet…); loss of assumptions about the world (the world does not make sense after all) *Manifestations of grief:* denial, anger, anxiety, sadness… *Manifestations of acceptance:* face the challenge, learn to prioritize, maintain a positive perspective, be more compassionate *Relationship dynamics* Peer relationships influenced psychological well-being Medication–peer-tug-of-war: negative reactions to the injections favoured the loss experience, positive reaction prompted acceptance Shifting friendships: losing friends, deepening old relationships, make new friends Finding supportive relationships: rely to a small group of positive and supportive friends Dealing with others’ worry: overprotection favours grief Talking about MS: despite cautiousness, disclosing the disease favours acceptance Acting normal: hide the diagnosis to maintain normalcy and avoid rejection
Experiences of children and adolescents living with multiple sclerosis. Boyd et al., 2005	Experiences of children and adolescents living with MS	*Learning the diagnosis: * fear, sadness, relief. Concerns about possible death *Noticing the differences: * symptoms and medication limiting everyday life *Staying the same: * the interviewed patients described a rather normal life, despite the difficulties, and did not feel changed by MS *Coping with MS: * stressors: intermittent symptoms, medication, being treated differently, missing school, parental worry, potential disability strategies: stay positive, identify role models with MS or other conditions, remain busy Unhealthy strategies can include denial, manipulation, unhealthy habits, etc. *Gaining support:* all patients refer to someone as really supportive *Dealing with treatment:* injection discomfort, bruises, side effects *Changing relationships:* feeling closer to the family; hide symptoms to prevent patients’ worry; received favouritism; manipulate; test friendships through diagnosis disclosure *Peer response:* usually positive, some experiences of exclusion from activities *Disclosing the diagnosis:* be selective *Effect learning:* learning problems, absenteeism *Looking toward the future:* move forward and be hopeful *Advice to peers with MS:* remain positive. Take care of yourself; medication is needed; seek support from others
